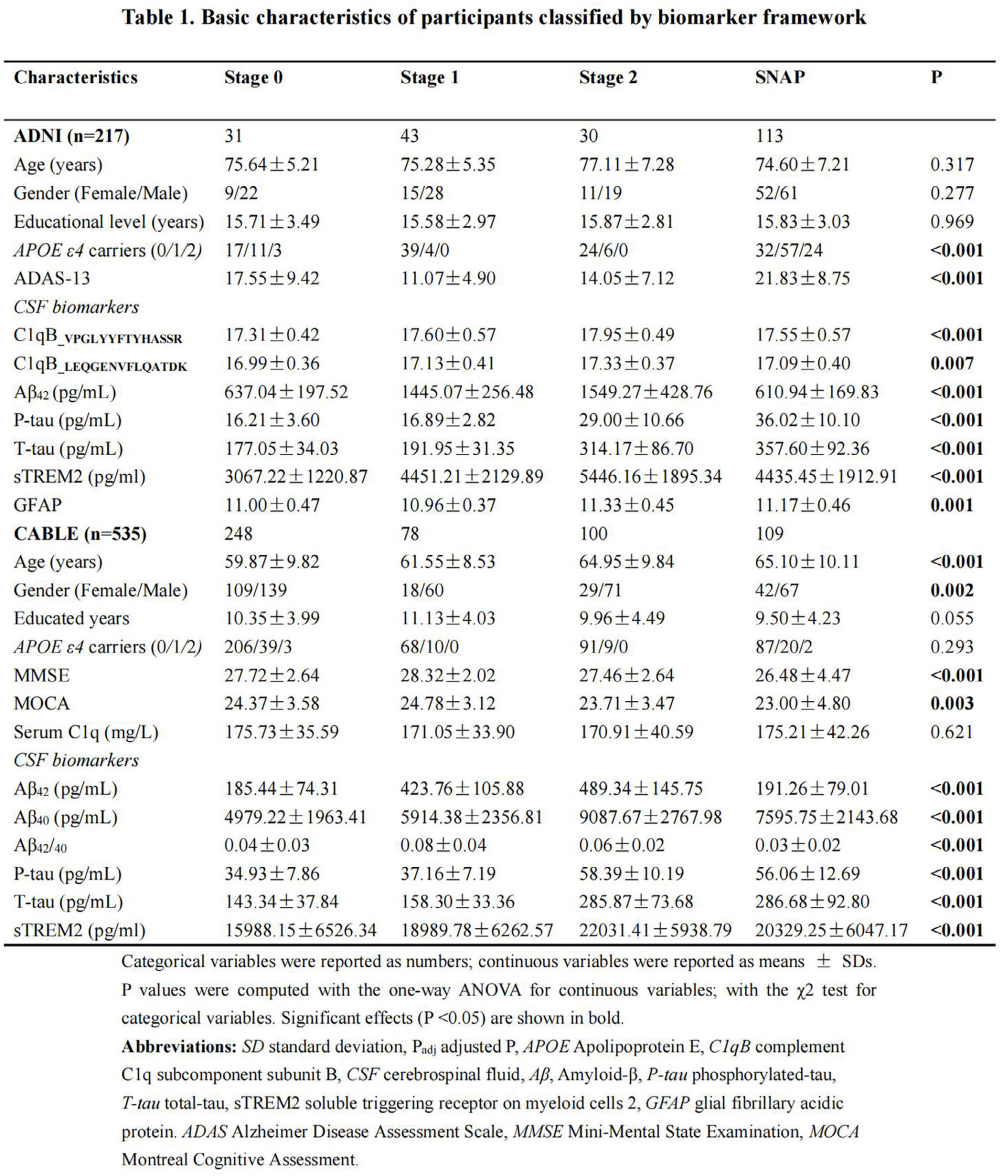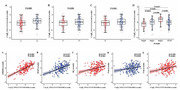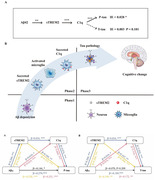# Complement C1q is Associated with sTREM2, GFAP and Alzheimer’s Pathology: the ADNI & CABLE Study

**DOI:** 10.1002/alz.088657

**Published:** 2025-01-09

**Authors:** Ze‐Hu Sheng, Zhibo Wang, Lingzhi Ma, Zuo‐teng Wang, Fan Guo, Lan Tan

**Affiliations:** ^1^ Department of Geriatrics, The First Affiliated Hospital of Chongqing Medical University, Chongqing Medical University, Chongqing, Chongqing China; ^2^ Department of Neurology, Qingdao Municipal Hospital, Qingdao University, Qingdao, Shandong China; ^3^ Xuanwu Hospital, Capital Medical University, Beijing, Beijing China; ^4^ Qingdao university, Qingdao China; ^5^ Qingdao Municipal Hospital, Qingdao University, qingdao, Shandong China; ^6^ Department of Neurology, Qingdao Municipal Hospital, Qingdao university, Qingdao, Shandong China; ^7^ Qingdao Municipal hospital, Qingdao university, Qingdao, Shandong China

## Abstract

**Background:**

Complement C1q, the initiator of the classical pathway of the complement system, is activated during Alzheimer’s disease (AD) development and progression and is especially associated with the β‐amyloid and tau pathology. However, whether C1q influences AD pathology by modulating glial cell communication is unclear.

**Method:**

Alzheimer’s Disease Neuroimaging Initiative (ADNI, N = 217) was used to explore the association between cerebrospinal fuid (CSF) C1q, soluble triggering receptor expressed on myeloid cells 2 (sTREM2), Glial fbrillary acidic protein (GFAP), and AD biomarkers. Chinese Alzheimer’s Biomarker and LifestylE (CABLE, N = 535) study was used to explore the association between plasma C1q, CSF sTREM2, GFAP, and AD biomarkers. Causal mediation analysis was conducted through 10,000 bootstrapped iterations to explore the mediating effect of sTREM2/GFAP on association between C1q and AD pathology.

**Result:**

We found that CSF C1q was positively associated with CSF sTREM2, GFAP and AD core biomarkers (Aβ42; phosphorylated‐tau, p‐tau; and total tau, t‐tau) at baseline, and was also significantly related to CSF t‐tau and ADAS‐13 in longitudinal analysis. Moreover, mediation analysis revealed that CSF C1q modulated the level of CSF sTREM2 and contributed to tau pathology (as measured by CSF p‐tau/ t‐tau). In addition, Aβ pathology (as measured by CSF Aβ42) affected the development of tau pathology (as measured by CSF p‐tau) partly by modifying the levels of CSF C1q and CSF sTREM2. Furthermore, the plasma C1q was also significantly associated with CSF sTREM2.

**Conclusion:**

Our fndings provide evidence to suggest that Complement C1q is linked to sTREM2 and mediates the correlation between Aβ pathology to tau pathology. It indicates that Complement C1q may be a major factor in the spreading from Aβ pathology to tau pathology in AD.